# Estrogen Receptor, Progesterone Receptor, and HER2 Receptor Markers in Endometrial Cancer

**DOI:** 10.7150/jca.41943

**Published:** 2020-01-16

**Authors:** Caifeng Wang, Davis A. Tran, Melinda Z. Fu, Wei Chen, Sidney W. Fu, Xu Li

**Affiliations:** 1 Emergency Department, Clinical Laboratory, Center for Translational Medicine, the First Affiliated Hospital of Xi'an Jiaotong University, Shaanxi, China.; 2 Department of Medicine (Division of Genomic Medicine), and Department of Microbiology, Immunology and Tropical Medicine, The George Washington University School of Medicine and Health Sciences, Washington, DC, USA.

**Keywords:** Endometrial cancer, ER, PR, HER2

## Abstract

**Background:** Endometrial cancer (EC) is a major gynecologic adenocarcinoma that arises from the endometrium. While the incidence of EC is on the rise worldwide, survivorship and clinical advancement have considerably lagged compared to other cancers. Given the sensitive nature of the endometrium and its high expression of hormone receptors, hormonal therapy has become a favorable alternative treatment compared to highly toxic chemotherapeutics and radiation therapy.

**Methods:** Clinical samples from patients diagnosed with EC were obtained. ER and PR staining were performed according to the S-P kit, and HER2 staining was carried out according to the UltrasensitiveTM S-P immunohistochemistry kit protocol. Chi-square analysis was conducted using the SPSS. P-values of less than 0.05 were taken as an *a priori* value for statistical significance.

**Results:** Immunohistochemical (IHC) analysis showed the overall positive expression rates of ER, PR, and HER2 to be 59.8%, 75.0%, and 71.1%, respectively. Significant co-expression was found among all three receptors, suggesting a cooperative, synergistic effect. More importantly, we found that ER expression was correlated with FIGO staging and cervical invasion, whereas PR expression was associated with histologic type. No clinicopathologic features were correlated with HER2 expression, but HER2 positivity was inversely associated with the degree of HER2 overexpression.

**Conclusions:** These results suggest that EC is a heterogeneous disease that may not conform to traditional, prototypically defined subtypes. The status of ER, PR, and HER2 receptors may have the potential to serve as prognostic indicators for EC, but further analysis is needed to ascertain their prognostic significance.

## Introduction

Endometrial cancer (EC) is a major gynecologic adenocarcinoma that arises from uninterrupted endometrial proliferation due to unopposed estrogen stimulation; predominantly in women of postmenopausal age 1-3. Worldwide, EC is the sixth most commonly diagnosed cancer in females, with the highest prevalence found among developed nations in North America as well as Northern and Eastern Europe 1, 4. In the United States and Canada, EC stands as the most common gynecologic cancer, whereas in China, EC is second only to cervical cancer 4, 5, 6. The incidence rate of EC is on the rise in 26 populations globally, with the greatest increase in Asia and Africa, largely due to their rapid socioeconomic transition 1.

In the United States, EC is one of few malignancies with increasing incidence and mortality. Between 1999-2016, EC incidence rose 0.7% per year, while mortality had a greater increase of 1.1% per year 7. Paradoxically, the survivorship for EC has worsened in the last four decades. The five-year survival rate between 1975 to 2014 has fallen from 86.9% to 82.7% (p<.05) and as of 2019, has decreased to 81.2% 5, 8. In China, there has been a significant rise in EC incidence 9. While data at the national level is not available, the most recently cited five-year survival rate for one registry is 55.1%, which is comparatively lower to the age-adjusted survival rate of 67% among developing nations 6, 9, 10.

Traditionally, EC lesions have been classified into two distinctive pathogenetic subtypes that differ in histological and molecular characteristics as defined by Bokhman 11. Type I consists of estrogen-dependent and low-grade lesions of endometrioid morphology with high mutations in *PTEN* 2, 4, 12. Type I tumors are more common (80% of cases) and are considered less aggressive, often found in early stage according to the Federation of Gynecology and Obstetrics (FIGO stage I-II), with high positivity for ER and PR as indicators of favorable prognosis 13. Type II is rarer and contains high-grade lesions of serous or clear-cell histology with frequent mutations in *TP53* and high expression and/or amplification of HER2 2, 14*.* Prognosis with type II tumors is generally poor with a higher chance for recurrence, as there is a predilection for deep myometrial invasion with more advanced stages (FIGO stage III-IV). Five-year overall survival for high-grade lesions amass only to 17%, with limited options beyond chemotherapy 15. While such classification on the basis of clinical, histological, and molecular features provides a powerful framework to derive potential prognostic markers for EC, the increasing heterogeneity within and overlap between type I and type II cancers is gaining more recognition 4. Thus, caution must be taken when determining the prognostic significance of hormone receptor status based solely on the dichotomous Bokhman classification.

Aside from clinical and pathological characteristics, endocrine markers in the form of ER, PR, and HER2 are particularly attractive as prognostic markers for EC given their direct involvement in the normal regulation and maintenance of endometrial health 16. In the regular progression of the menstrual cycle, the lining of the uterus is subject to a pair of steroid hormones, estrogen and progesterone, that each exerts an opposing effect on the endometrial glandular epithelium 13, 17, 18. In particular, estrogen has a mitogenic effect that drives the proliferation of the endometrial epithelium via ER. Left unopposed, estrogen can lead to the rapid onset of endometrial hyperplasia and consequently, the development of EC. Progesterone, however, acts as an antagonist to estrogen by downregulating ER expression, inhibiting active cell division, and promoting cell differentiation through PR 18, 19.

As the endometrium expresses both ER and PR, the lining of the uterus is highly sensitive to hormone activity 18. Therefore, any shift to the endocrine balance in favor of high estrogen level will ultimately stimulate oncogenesis. Such overexposure to estrogen arises in the majority of type I tumors, which also becomes a high risk factor among women undergoing estrogen-only hormonal therapy, using tamoxifen as adjunct therapy for breast cancer, facing obesity as adipose tissue releases estrone, which is converted into estradiol in the uterus, or suffering from PCOS (polycystic ovary syndrome) 20, 21.

HER2, a well characterized oncogene in the pathogenesis of breast cancer, has also been implicated as a potential biomarker for type II tumors 22. In brief, overexpression of HER2 results in sustained cell proliferation via constitutive activation of the kinase domain in a ligand-independent manner 14. While HER2 expression is mostly associated with a poor prognosis in type II lesions, recent studies suggest 1-47% of HER2 overexpression is also found in advanced and recurrent type I endometrioid carcinomas 14. Given the need for receptors for hormones to successfully exert their downstream effects on the endometrium, hormone receptor status may therefore be a valuable prognostic marker for EC development and progression.

Staged surgery is currently the only primary treatment for EC, followed by adjuvant chemotherapy or radiation therapy 2, 23. As EC patients are generally of older age and are more likely to have comorbidities, the aversive side effects from chemotherapy are unable to justify the low response rate (less than 20%) 15, 24. Furthermore, 14% of all EC cases are found in women of child-bearing age 18, 25. As such, hormone therapy is an attractive treatment not only for its lower toxicity profile, but also for its use in preserving fertility among younger EC patients.

Even though there is a number of studies on the potential use of hormone receptor status to refine outcome predictions for EC, the prognostic significance of hormone receptor profiles in EC remains unclear 26. Even fewer studies are available on receptor status in Chinese population. In an attempt to characterize the prognostic value of hormone receptors on a global level, Zhang et al. 13 conducted a systematic review and meta-analysis for the expression rate of ER, PR, and HER2 in EC, with 48, 38, and 16 studies, respectively. Of those selected for inclusion, only 3 studies pertained specifically to the Chinese population regarding ER and PR status. Moreover, studies involving HER2 expression in Chinese women were not included. Therefore, the present study seeks to investigate the hormone receptor status of ER, PR, and HER2, both independently and collectively, in a cohort of Chinese women. Receptor expression rates are also analyzed for associations to clinicopathologic characteristics to determine the prognostic value of receptor status in EC.

## Materials and Methods

### Study Participants

In total, this study consisted of 204 women of Chinese descent that were residents of or within the neighboring regions of Shaanxi province between June 2000 and February 2007. Of the 204 participants, 89 were diagnosed with EC while 115 served as controls, presenting with no current or history of cancer and systemic diseases. The women varied in age, from 30-80 years with a median age of 60 years. Participants took a self-administered study questionnaire requesting relevant medical and social history.

### Sample Collection and Classification

Clinical samples from patients diagnosed with EC were obtained between June 2000 and February 2007, after radical surgery or total hysterectomy in the Department of Gynecology and Obstetrics at the First Affiliated Hospital of Xi'an Jiaotong University, in Xijing Hospital of the Fourth Military Medical University, and at the Shaanxi Provincial People's Hospital and Xi'an Fourth Hospital within Shaanxi province. The patients varied in age, between 30 to 80 years, with a median of 60 years. The samples were matched with a control population with an age range of 31 to 79 years, with a median age of 46 years. The study protocols were approved by our institution's ethics committee and all participants were consented.

EC patient samples were then subject to analysis and classification based on histologic subtype (type I or type II), surgical staging (I-IV) according to the Federation of Gynecology and Obstetrics (FIGO) 2009 criteria, histologic grade (G1-G3), and invasive status to the myometrium and cervix.

### Immunohistochemistry assay

ER and PR staining were performed according to the S-P kit (Beijing Zhongshan Jinqiao Biotechnology Co., Ltd.), and HER2 staining was carried out according to the UltrasensitiveTM S-P immunohistochemistry kit protocol (Maixin Biotechnology Development Co., Ltd.). The S-P immunohistochemistry kit uses a biotin-labeled secondary antibody and a streptavidin-linked peroxidase, and the substrate pigment mixture to determine antigens in the nucleus. The PBS solution was used as a negative control, and the known positives provided by the company were used as positive controls.

The overall expression levels of ER (n=82), PR (n=80) and HER2 (n=76) were detected in formalin-fixed and paraffin embedded (FFPE) tissue by immunohistochemistry (IHC) assay in 4-mm thick sections. In brief, samples were deparaffinized with xylene and rehydrated with 0.3% hydrogen peroxide (H_2_O_2_) solution. After immersion in a 10^-3^ M sodium citrate buffer, the slides were pressurized for 1 h, after which they were cooled and washed with phosphate buffered saline (PBS). Tissues were then incubated in primary antibodies overnight at 4ºC, retrieved, and washed with PBS. Following incubation in biotinylated anti-mouse secondary antibodies and streptavidin for 30 mins at room temperature, the samples were stained with alkaline phosphatase enzyme conjugates. Comparative staining was conducted with hematoxylin. Two pathologists with no prior information concerning scored clinical variables interpreted all immunohistochemical staining results. A cut-off value of 10% for positively stained cells per ten high-power fields was used in the classification of the ER, PR and HER2 protein expression.

### Statistical Analysis

Chi-square analysis was conducted using the SPSS. P-values of less than 0.05 were taken as an *a priori* value for statistical significance.

## Results

### ER Expression & Association with Clinicopathologic Features

**Figure 1:**
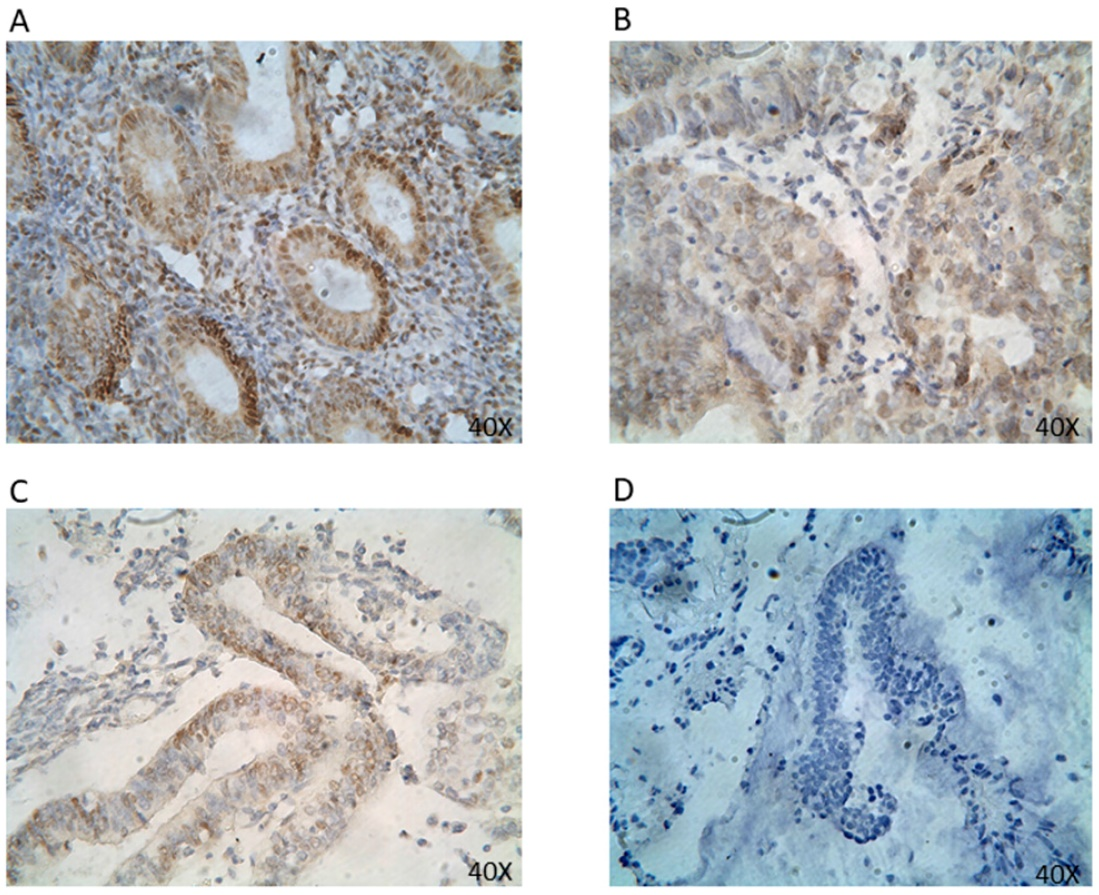
** ER protein expression in EC tissues by IHC (40x).** Representative EC FFPE tissues with IHC staining of ER. IHC shows nuclear stain positivity for ER expression in the cell nucleus. IHC results are shown at 40x magnification.

The expression rate for ER positivity was 59.8% (*Table 1*). ER proteins were found primarily expressed in the nucleus (*Figure 1*). ER expression showed statistically significant correlation with FIGO staging, histologic grade, and cervical invasion (p<0.05) (*Table 2*). In particular, there was a significant association between ER positivity and FIGO stage I (69.1%). A significant difference in ER expression among various FIGO stages was observed. ER expression further showed a significant association with histologic grade G1 (74.2%) and cervical invasion (36.8%). There was no significant association between ER status with either histologic type or myometrial invasion.

### PR Expression with Clinicopathologic Features

**Figure 2:**
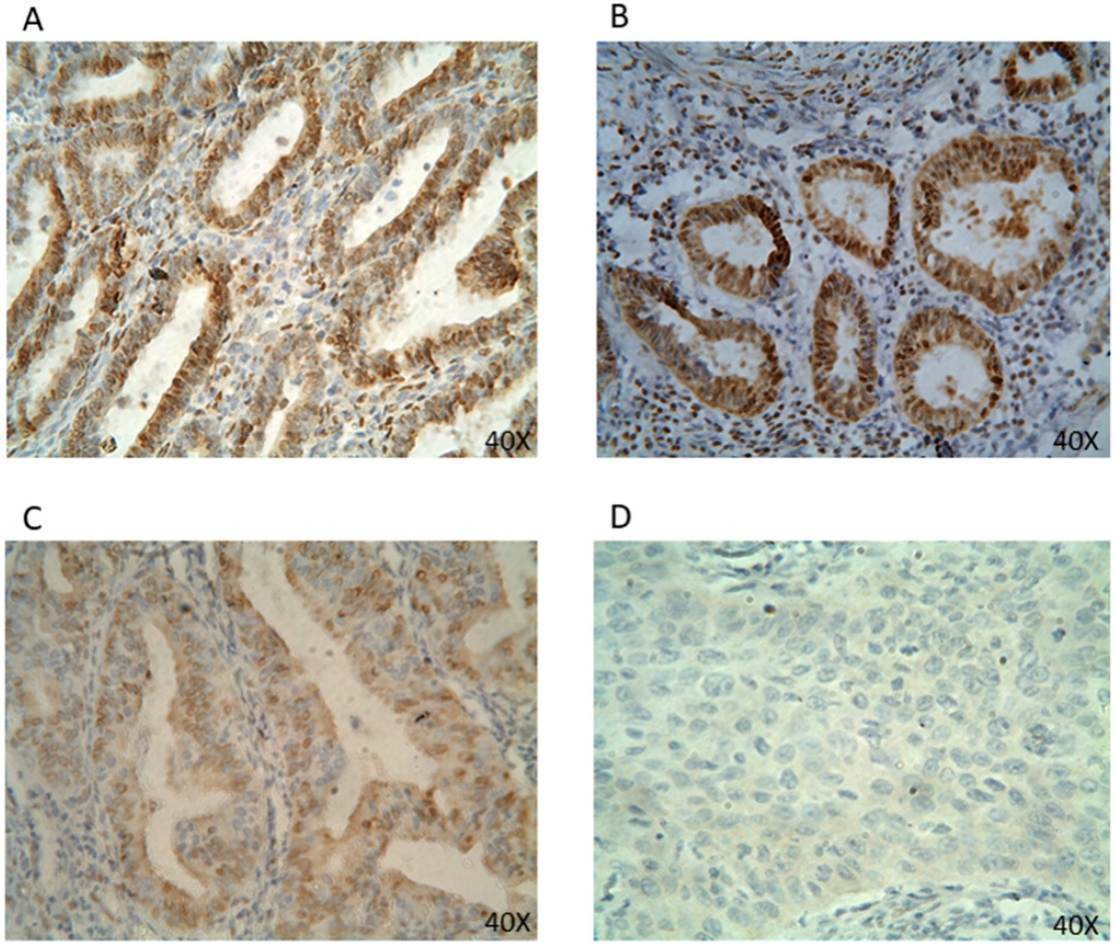
** PR protein expression in EC tissues by IHC (40x).** Representative EC FFPE tissues with IHC staining of PR. IHC show nuclear stain positivity for PR expression in the cell nucleus. IHC results are shown at 40x magnification.

The expression rate for PR positivity was 75.0% (*Table 1*). PR protein was primarily expressed in the cell nucleus (*Figure 2*)*.* PR expression was correlated to the difference in histologic type (77.6% type I vs. 25.0% type II) and FIGO stage I (81.1%) with statistical significance (*Table 2*). PR expression showed no significant correlation in either histologic gradation or invasion status of the myometrium or cervix.

### HER2 Expression in Histologic Types of EC and Association with Clinicopathologic Features

**Figure 3:**
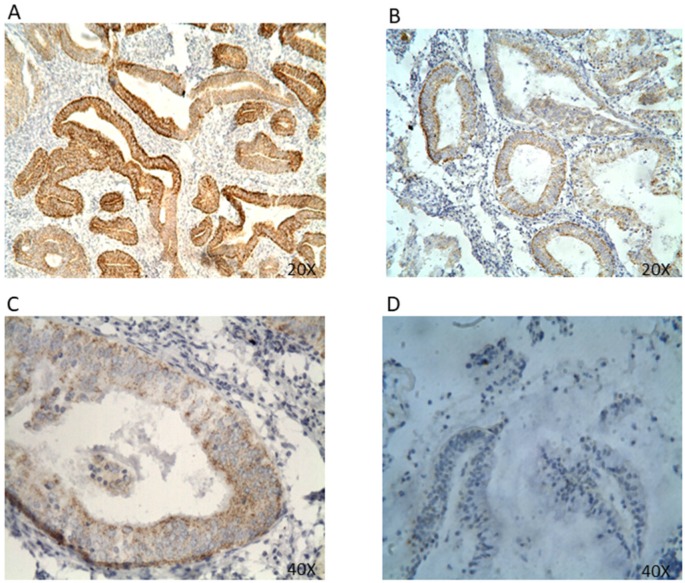
** HER2 protein expression in EC tissues by IHC (A&B 20x; C&D 40x).** Representative EC FFPE tissues with IHC staining of HER2. HER2 expression was mainly found expressed in the cell membrane and cell cytoplasm. (A&B) IHC results are shown at 20x magnification; (C&D) IHC results are shown at 40x magnification.

The positive expression rate for HER2 was 71.1%, with a strong overexpression rate (3+ IHC) of 2.8% in endometrioid endometrial carcinoma (EEC) (*Table 1 & Table 3*)*.* HER2 protein was expressed in both cytoplasm and membrane (*Figure 3*)*.* HER2 expression showed no significant correlation to any clinicopathologic parameter or histologic classification. However, when analyzed in endometrioid EC, there was a significant negative association between HER2 positivity and the degree of HER2 expression (p<0.05) (*Table 3*). HER2 status was also investigated based on EC's distinct variants, which showed positive HER2 expression in 72.1% cases of endometrioid endometrial carcinoma (EEC), 25.5% non-endometrioid endometrial carcinoma (NEC), 50% clear cell carcinoma (CCC), 0% uterine papillary serous carcinoma (UPSC), and 0% squamous carcinoma (SC) (*Table 3*).

### Co-expression of ER, PR, & HER2

The co-expression of each pair of the three hormone receptors was analyzed. Each pair showed a positive and significant correlation (p<0.01). Specifically, out of 54 HER2-postive cases, 70.4% was found to have positive co-expression of HER2 and ER (p <0.01) (*Table 4a*). In addition, in 55 HER2-positve cases, 83.6% was found to have co-expression between HER2 and PR (p=0.01) (*Table 4b*). Lastly, in 45 ER-positive cases, 95.6% was found to have co-expression between ER and PR (p=0.01) (*Table 4c*).

## Discussion

### Independent ER & PR Receptor Status in EC

Given the endometrium's high sensitivity to steroid hormones and the significant impact hormones have in modulating endometrial growth, molecular tumor classification based on receptor status is suspected to play an important prognostic role in the management and treatment of EC. The presence of steroid receptors ER and PR, in particular, have been considered to be associated with favorable outcomes in the majority of type I tumors 2, 13, 17, 19, 27; however, its prognostic significance is not universally accepted and remains unclear. In this study, both ER and PR protein were expressed in the cell nucleus (*Figure 1 & 2*). The positive expression rates for ER and PR were 59.8% and 75.0%, respectively (*Table 1*). Such figures are in agreement with previous studies 23, 26-29. Among them, the most recent figures by Tomica, Ramic 28 reported a positive rate of 65.2% for ER expression and 59.4% for PR expression in a Caucasian population. Interestingly, while our data on ER and PR receptor status resonates with those found in a Caucasian population, our positive rates are lower in comparison to those reported in a Chinese cohort by Shen et al. 17, which showed an overall rate of 85% for both ER and PR expression. Such disparity may be the result of the sequential loss of receptors in disease progression 26, as patients were neither stratified by the year of diagnosis, nor was the duration of EC analyzed in either study.

### The Independent Association of ER & PR Receptor Status with Clinicopathologic Features

Bokhman classification, FIGO surgical staging, histologic grade, and invasion status are well accepted prognostic factors that show a significant association with patient survival in EC 28, 30. Hormone receptor status is also correlated with the aforementioned clinicopathologic traits. Specifically, the loss of ER and PR status is associated with EC lesions that are designated as type II, of higher tumor grade, and are more prone to deep myometrial invasion 2, 3, 17, 28, 29, 31. In this study, the statistically significant difference in FIGO staging by immunohistochemistry (IHC) further lends support to the notion that a decrease in ER expression is correlated to more advanced stages of EC (p<0.05) (*Table 2*). A similar inverse trend is observed with PR status and FIGO stages. However, except for stage I and PR expression, the difference among stages are statistically insignificant with PR status. This suggests ER expression is a better indicator of FIGO staging compared to PR status, at least among Chinese women found in this study.

While ER expression was not correlated with histologic type, PR receptor status did exhibit a significant association in the difference between histologic types I vs. type II (*Table 2*). This suggests that PR status is a more reliable predictor of patient outcome when compared to ER status when histologic type is analyzed in isolation. However, the limitations of a smaller sample size of cases for type II tumors, advanced staging, and lesions of higher grade must also be recognized.

Shen et al. [17]noted that while ER and PR status were higher among type I patients in their Chinese cohort as expected, the majority of type II patients also contained notable levels of ER and PR expression. However, we found PR expression in only 25% of type II patients compared to 77.6% of type I patients (*Table 2*). The differences observed may therefore suggest EC is a heterogeneous disease that may have attributes outside of those historically defined by the dichotomous Bokhman classification system 12, 22. It is noteworthy to recognize that in light of substantial heterogeneity within and between type I and type II cancers, Bokhman's type I and type II are not part of the formal FIGO staging or risk stratification processes, and therefore has no clinical utility as discussed 4. Not all endometrioid lesions act in the same prototypical type I fashion and vice versa 2. In fact, a number of type I lesions are of high grade and appear to be as aggressive as traditionally characterized type II lesions 2. More recently, the Cancer Genome Atlas (TCGA) focused on molecular subgroups of EC and identified how endometrioid lesions, classically designated as type I, exhibited heterogeneity within the group as cases varied in mutation load, grade, and prognosis 4, 12.

Although the difference in histologic grade and invasion status may be statistically insignificant, a cogent negative trending association is apparent between the loss of both ER and PR receptors in higher grade tumors, myometrial invasion, and cervical invasion (*Table 2*). Furthermore, ER expression levels in histologic grade G1 and cases in which cervical invasion had occurred were both of statistical significance (*Table 2*). Taken together, our data is in concordance with the general association between independently high ER and PR status with a favorable prognosis of EC 13.

### HER2 Receptor Status in EC

In a similar fashion to ER and PR, HER2 has been investigated for its prognostic value in endometrial cancer, but its prognostic significance also remains controversial 14, 22. Contrary to ER and PR expression found predominantly in the cell nucleus, HER2 protein was found in both the cell cytoplasm and membrane (*Figure 3*)*.* Furthermore, we identified HER2 expression in 71.1% of all EC cases, which is comparable to the reported positive rate of 71.1% by Wang et al. 32 in a Japanese cohort as opposed to 7.14% by Waqar et al. 3 among Pakistani women (*Table 1*). However, much attention concerning HER2 status has been focused on its association with more aggressive type II lesions, particularly in uterine papillary serous carcinoma (UPSC) 2, 14, 19, 22. In the literature, expression of HER2 varied over a range from 14 to 80% 2, 14, 33-35. Interestingly, our IHC analysis did not detect HER2 expression in UPSC (*Table 3*). Rather, endometrioid endometrial carcinoma (EEC) contained the highest level of HER2 expression at 72.1%, with a strong protein overexpression rate (3+ IHC) of 2.9% *(Table 3)*. While IHC data on HER2 status in UPSC is inconsistent with the majority of prior reports, HER2 positivity in EEC has been documented to appear within a range of 1% to 47% in advanced-stage type I lesions 14 in spite of some disagreement 33. It is also important to note that the lack of HER2 detection may be attributable to the small number of UPSC cases (n=1) compared to EEC (n=68). Furthermore, there was a statistically significant difference in the inverse relationship between decreasing HER2 positivity rates and the degree of HER2 expression or IHC score (p<0.05) (*Table 3*). The positive rates for non-endometrioid endometrial carcinoma (NEC), clear cell carcinoma (CCC), and squamous carcinoma (SC) were 25.0%, 50%, and 0%, respectively. Nevertheless, such positivity for HER2 in EEC (72.1%) further underscores both the heterogeneity and complexity of EC.

### Association of HER2 Receptor Status with Clinicopathologic Features

While our data shows HER2 expression did not significantly correlate with any histologic subtype or clinicopathologic characteristic, it is still noteworthy to analyze the trends between HER2 status and clinicopathologic features (*Table 2*). Zhang et al. 13 suggested that high HER2 positivity is associated with an unfavorable prognosis, whereas high ER and PR positive rates are good predictors of patient survival. Surprisingly, HER2 was found to exhibit low expression in both high-grade tumors and in positive cases of cervical involvement, a trend similarly seen in ER and PR receptor status (*Table 2*). Additionally, HER2 status was more associated with type I tumors than type II (72.1% vs. 25.0%). Yet, HER2 maintained high positivity for advanced FIGO stages and myometrial involvement, albeit statistically insignificant. Such results suggest the importance of adopting a comprehensive approach to guide therapeutic and clinical decisions as opposed to relying on any one clinicopathologic feature.

### Co-expression of ER, PR, and HER2

Aside from analyzing each hormone receptor status independently, the co-expression of hormone receptors was also considered (*Table 4a, 4b, 4c*). Specifically, out of 54 HER2-postive cases, 70.4% was found to have concurrent expression of both HER2 and ER, whereas 83.6% of cases showed co-expression of HER2 and PR with statistical significance (*Table 4a & Table 4b*). Finally, in 45 ER-positive cases, 95.6% was found to have a significant level of both ER and PR expression (*Table 4c*). Such findings regarding the co-expression of ER and PR is concordant with the literature, as estrogen has been shown to regulate PR expression and this may explain how prolonged use of progestin therapy confers resistance 13, 18.

In terms of HER2 co-expression, however, Mariani et al. 19 noted an inverse correlation between HER2 and PR status, suggesting HER2 overexpression may result in the downregulation of PR, thereby giving rise to a hormone-independent growth mechanism that is highly characteristic of type II EC. Similar to Backe et al. 36 and Niederacher et al. 37, we were unable to replicate this finding, as only 16.4% of HER2-positive cases showed negative PR expression compared to 83.6% of cases that were positive for both HER2 and PR (*Table 4b*). Alternatively, Samsonova et al. 38 found no significant correlation between HER2 expression and the expression of ER and PR. Interestingly, our data suggests all three receptors are co-expressed and that ER, PR, and HER2 may therefore exert a synergistic effect in the course of EC pathogenesis and progression. The mechanism of cooperative action, however, awaits further investigation.

In conclusion, among our cohort of Chinese women, we found the overall positive expression rates of ER, PR, and HER2 to be 59.8%, 75.0%, and 71.1%, respectively (*Table 1*). When compared to a similar Chinese cohort, there were varied differences in receptor status, further supporting the notion that there are various subtypes of EC that do not fit in the traditionally dualistic classification of EC lesions. Furthermore, HER2 expression was higher in type I lesions and the positive rate was comparable to those found for ER and PR expression (*Table 2*).

Independent receptor status was also associated with some, but not all clinicopathologic features (*Table 2*). ER expression was associated with FIGO staging, while PR expression correlated with histologic type with statistical significance. This suggests ER receptor status may be a better indicator of FIGO staging while PR expression is more correlated to histologic type. HER2 receptor status, known for its association with the more aggressive phenotype of EC, surprisingly demonstrated low expression in UPSC (0%) and in all type II lesions (25%) compared to the high expression found in EEC (71.2%) (*Table 3*). Such disparity against the literature may be attributable to the small number of UPSC cases. A larger sample size and continued investigation is necessary to better elucidate the association between HER2 receptor status and UPSC. The present study further suggested that all three hormone receptors are co-expressed and most likely exert a cooperative effect (*Table 4a, 4b, 4c*).

Our data further underscores the need to view EC as a dynamic and heterogeneous disease. When selecting treatment, it is also of vital importance for clinicians to consider the full breadth of clinicopathologic features while analyzing each patient on an individual basis, as hormone receptor status may vary for different clinicopathologic features. Further investigation focusing not only on associations between various receptor statuses and types of EC, but also into the mechanistic role that various receptors play in the pathogenesis of EC is further warranted to fully understand the prognostic significance of hormone receptor expression in both the progression and management of EC.

## Figures and Tables

**Table 1:** Overall Expression of ER, PR, and HER2

ER Expression n=82		PR Expression n=80		HER2 Expression n=76	
Positive (%)	Negative (%)	Positive (%)	Negative (%)	Positive (%)	Negative (%)
49 (59.8)	33 (40.2)	60 (75.0)	20 (25.0)	54 (71.1)	22 (28.9)

**Table 2:** Association of ER, PR, and HER2 Expression with Clinicopathologic Features

Clinicopathologic Feature	ER Expression					PR Expression					HER2 Expression			
	Cases	Positive (%)	Negative (%)	p-value		Cases	Positive (%)	Negative (%)	p-value		Cases	Positive (%)	Negative (%)	p-value
Bokhman Subtype	80			0.628		80			0.046*		72			0.202
Type I	76	48 (63.2)	28 (36.8)			76	59 (77.6)	17 (22.4)			68	49 (72.1)	19 (27.9)	
Type II	4	2 (50)	2 (50)			4	1 (25.0)	3 (75.0)			4	1 (25.0)	3 (75.0)	
														
FIGO Staging	82			0.047*		80			0.094		75			0.913
I	55	38 (69.1)	17 (30.9)	0.022*		53	43 (81.1)	10 (18.9)	0.057*		48	34 (70.8)	14 (29.2)	0.912
II	18	7 (38.9)	11 (61.1)	0.254		18	10 (55.6)	8 (44.4)	1		18	13 (72.2)	5 (27.8)	1
III & IV	9	4 (44.4)	5 (55.6)	1		9	7 (77.8)	2 (22.2)	0.406		9	7 (77.8)	2	1
														
Histologic Grade	80			0.097		78			0.527		74			0.783
G1	31	23 (74.2)	8 (25.8)	0.031*		29	24 (82.8)	5 (17.2)	0.265		27	19 (70.4)	8 (29.6)	0.591
G2	39	19 (48.7)	20 (51.3)	0.441		38	27 (71.1)	11 (28.9)	0.660		38	29 (76.3)	9 (23.7)	1
G3	10	6 (60.0)	4 (40.0)	0.725		11	8 (72.7)	3 (27.3)	1		9	6 (66.7)	3 (33.3)	0.674
														
Invasion of Myometrium	82			0.766		80			0.162		73			0.916
Invasion>50%	16	9 (56.3)	7 (53.7)	0.848		16	11 (68.8)	5 (31.2)	1		12	9 (75.0)	3 (25.0)	1
Invasion≤50%	56	33 (58.9)	23 (41.1)	0.683		55	39 (70.9)	16 (29.1)	0.123		52	37 (71.2)	15 (28.8)	1
Non-invasion	10	7 (70.0)	3 (30.0)	0.728		9	9 (100)	0 (0)	0.097		9	6 (66.7)	3 (33.3)	1
														
Invasion of Cervix	81					80			0.083		73			0.766
Invasion	19	7 (36.8)	12 (63.2)	0.016*		19	11 (57.9)	8 (42.1)			16	11 (68.8)	5	
Non-invasion	62	42 (67.7)	20 (32.3)			61	48 (78.7)	13 (21.3)			57	41 (71.9)	16	

**Table 3:** Distribution of HER2 Expression in Histopathological Subtypes of Endometrial Cancer

		Histopathological Subtype			
Degree of HER2 Expression (IHC Score)	Case n=72 (%)	EEC* n=68 (%)	UPSC n=1 (%)	CCC n=2 (%)	SC n=1 (%)
0	22 (30.6)	19 (27.9)	1 (100)	1 (50)	1 (100)
1+	39 (54.2)	38 (55.9)		1 (50)	
2+	9 (12.5)	9 (13.2)			
3+	2 (2.8)	2 (2.9)			
Positive Rate (%)	69.4	72.1	0	50	0

**Table 4a:** Co-expression of HER2 and ER

		ER Expression		Positive Rate (%)	X^2^	OR (95%CI)	P-value
**Cases**	**HER2** **Expression**	**(-)**	**(+)**		7.566	4.156 (1.459-11.839)	0.006^*^
22	**(-)**	14	8	36.4			
54	**(+)**	16	38	70.4			

**Table 4b:** Co-expression of HER2 and PR

		PR Expression		Positive Rate (%)	X^2^	OR (95%CI)	P-value
**Cases**	**HER2** **Expression**	**(-)**	**(+)**		7.918	4.646 (1.523-14.173)	0.005^*^
21	**(-)**	10	11	52.4			
55	**(+)**	9	46	83.6			

**Table 4c:** Co-expression of ER and PR

		PR Expression		Positive Rate (%)	X^2^	OR (95%CI)	P-value
**Cases**	**ER** **Expression**	**(-)**	**(+)**		26.104	27.643 (5.691-134.267)	0.000*
32	**(-)**	18	14	43.8			
45	**(+)**	2	43	95.6			

## Abbreviations

estrogen receptor): ER
progesterone receptor): PR
human epidermal growth factor receptor 2): HER-2
endometrial cancer): EC
Immunohistochemistry): IHC
